# Case of Irregularity of Superior Cuspid

**Published:** 1901-01

**Authors:** H. S. Bascom


### CASE OF IRREGULARITY OF SUPERIOR CUSPID.[1]

BY DR. H. S. BASCOM.

Mr. President and Gentlemen,—The case of irregularity to which I have been invited to ask your attention to-night is not one involving several irregular teeth brought after weeks or months of labor to a successful finish, but, on the other hand, it is a case of one tooth erupted out of its proper position and being moved to a place where it would look as it ought to look. And although it has been worked upon for weeks and even months, it still is very far

[1] Read before The New York Institute of Stomatology.

from being in its proper position. The principal trouble was caused by an accident at the very beginning.

To be as brief as possible, the case is simply this: A young lady of sixteen called upon me with her mother to know what was best to do about the left upper cuspid, which was a little more than half erupted and was located almost directly over the lateral incisor. An examination of all the teeth showed that a well-nigh perfect articulation existed on the right side and including the left central, first and second left bicuspids, and first and second molars. The left lower cuspid was standing a little out of its proper position, so that the left upper lateral incisor shut inside the lower cuspid. The upper left lateral incisor and first bicuspid nearly touched each other.

Under the circumstances, what was simpler to do than to remove the left first bicuspid and move the cuspid into the place occupied by the bicuspid. This I suggested to be the better way, everything considered, as they did not care to interfere with the lower teeth.

I was at that time moving a right upper cuspid for a friend of this lady. They decided to wait a little time and have something done later, possibly to see how the other lady's tooth succeeded, which I am pleased to say did succeed to the satisfaction of all concerned. Therefore, I received a second visit from the would-be patient, and was asked to proceed as rapidly as safety would permit. An impression of both upper and lower teeth was taken, and models secured. On the upper plaster model I cut off the first bicuspid and proceeded to make my regulating device. A few days later, by appointment, the patient came in. Beta-eucaine was injected and the first bicuspid was extracted, but the root broke off just where the two small roots joined the main root (this was where the accident above referred to came in). Looking at this broken root, the two roots were demonstrated to be of an unusually small size. This was the first time I ever broke a tooth in extracting for correcting irregularity. I dismissed my patient to come the next day. In the mean time I sought for information in text-books and dental literature as to what was best to do, but I discovered nothing satisfactory. The next day I placed on the fixture, which was kept in active working order for a month, when I discovered that the second bicuspid and first molar on the left side had moved a little outward without apparently moving the cuspid. A new impression was taken and a new and stronger fixture was made. A month later the tooth had gone back a little more than one-sixteenth of an inch. This brought me to April 1.

In the mean time I had asked dentist after dentist for advice as to how to move that tooth, how to get rid of that root, but had received no satisfactory answer, when I decided to apply to Dr. Jackson, of this city. He replied that the fixture was correct, except to extend a wire back of the second molar, and thus get more anchorage. I therefore made a new fixture. He also suggested cutting out the root with large-sized pointed fissure burs with the dental engine, cutting it out thoroughly, including considerable of the alveolar process connected with it. This was just the information I wanted. Why had I not thought of it?

I immediately asked permission of my patient to remove that root, but nearly three weeks elapsed ere my request was granted. I procured a spear-shaped drill, a large-sized round excavating bur, and a few large-sized dentate pointed fissure burs. I found these burs to work best; of course they soon became cloyed, but a clean one was substituted; in this way the cutting was soon completed, and by the use of beta-eucaine was nearly painless. In using the burs, the gum where the tooth was extracted was cut away just enough to form a flap and brought over one side. The instrument was pushed into the place where the tooth was extracted (which had by this time become partially healed), and while revolving was pushed up to the broken root. The feel of the bur was easily to be noticed when the root, and also when the more porous process, was being cut. A week later the tooth gave more signs of moving. The good work was carried on until about July 1, when the tooth nearly touched the wire hook near the second bicuspid, and a change in the fixture must be made.

At this time I was informed the patient was going to the seashore, and could not conveniently see me but once or twice more; therefore I made a fixture which is little more than a retaining device. During July I saw her twice, and during August she went to the mountains, and I saw no more of her until September 10, when her teeth were in the condition as you see them on the model. The fixture had become somewhat loose and had not been worn for six weeks, therefore the cuspid had gone back some; and now she has gone to a boarding-school away from New Haven, and I fail to see how I will be able to finish my work.

